# Community-based progress indicators for prevention of mother-to-child transmission and mortality rates in HIV-exposed children in rural Mozambique

**DOI:** 10.1186/s12889-021-10568-4

**Published:** 2021-03-17

**Authors:** Laura Fuente-Soro, Sheila Fernández-Luis, Elisa López-Varela, Orvalho Augusto, Tacilta Nhampossa, Ariel Nhacolo, Edson Bernardo, Blanca Burgueño, Bernadette Ngeno, Aleny Couto, Helga Guambe, Kwalila Tibana, Marilena Urso, Denise Naniche

**Affiliations:** 1 Centro de Investigação em Saúde de Manhiça, Maputo, Mozambique; 2 Barcelona Institute for Global Health, Barcelona, Spain; 3 ISGlobal, Barcelona Institute for Global Health, Rossello, 132, 08036 Barcelona, Spain; 4 Instituto Nacional de Saúde, Maputo, Mozambique; 5 Manhiça District Health Services, Maputo, Mozambique; 6 Vanderbilt Institute for Global Health, Nashville, Tennessee USA; 7 Division of Global HIV and Tuberculosis, U.S. Centers for Disease Control and Prevention, Atlanta, USA; 8 Ministério da Saúde de Moçambique, Maputo, Mozambique; 9 Division of Global HIV and Tuberculosis, U.S. Centers for Disease Control and Prevention, Maputo, Mozambique

**Keywords:** HIV, MTCT, Mozambique, Africa, Mother-to-child transmission, HIV-prevalence

## Abstract

**Background:**

Eliminating mother-to-child HIV-transmission (EMTCT) implies a case rate target of new pediatric HIV-infections< 50/100,000 live-births and a transmission rate < 5%. We assessed these indicators at community-level in Mozambique, where MTCT is the second highest globally..

**Methods:**

A cross-sectional household survey was conducted within the Manhiça Health Demographic Surveillance System in Mozambique (October 2017–April 2018). Live births in the previous 4 years were randomly selected, and mother/child HIV-status was ascertained through documentation or age-appropriate testing. Estimates on prevalence and transmission were adjusted by multiple imputation chained equation (MICE) for participants with missing HIV-status. Retrospective cumulative mortality rate and risk factors were estimate by Fine-Gray model.

**Results:**

Among 5000 selected mother-child pairs, 3486 consented participate. Community HIV-prevalence estimate in mothers after MICE adjustment was 37.6% (95%CI:35.8–39.4%). Estimates doubled in adolescents aged < 19 years (from 8.0 to 19.1%) and increased 1.5-times in mothers aged < 25 years. Overall adjusted vertical HIV-transmission at the time of the study were 4.4% (95% CI:3.1–5.7%) in HIV-exposed children (HEC). Pediatric case rate-infection was estimated at 1654/100,000 live-births. Testing coverage in HEC was close to 96.0%; however, only 69.1% of them were tested early(< 2 months of age). Cumulative child mortality rate was 41.6/1000 live-births. HIV-positive status and later birth order were significantly associated with death. Neonatal complications, HIV and pneumonia were main pediatric causes of death.

**Conclusions:**

In Mozambique, SPECTRUM modeling estimated 15% MTCT, higher than our district-level community-based estimates of MTCT among HIV-exposed children. Community-based subnational assessments of progress towards EMTCT are needed to complement clinic-based and modeling estimates.

**Supplementary Information:**

The online version contains supplementary material available at 10.1186/s12889-021-10568-4.

## Introduction

Elimination of mother-to-child HIV transmission (EMTCT) is a critical milestone in ending the HIV epidemic globally by 2030. The World Health Organization (WHO) defines EMTCT as a rate of < 50 new pediatric HIV infections per 100,000 live births and a transmission rate < 5% in breastfeeding infants, maintained for at least 1 year [1]. In 2012, with the aim of reducing mother-to-child HIV transmission (MTCT), the WHO recommended Option B+, an approach that ensured universal lifelong treatment for pregnant women (regardless of CD4 count) and daily nevirapine or zidovudine for their children during the first 4–6 weeks of life regardless of whether or not they were breastfeeding [2]. Currently, prevention and surveillance of MTCT (PMTCT) for HIV-exposed children (HEC) includes early infant diagnosis conducted between 4 and 6 weeks of life and clinical follow-up until 18 months of life or until the end of breastfeeding. Although the global number of annual new infections in children (aged 0–14 years) has decreased from 230,000 to 160,000 and the associated HIV-associated child mortality from 170,000 to 100,000 deaths (2012–2018), the absolute numbers of annual cases remain elevated, especially in sub-Saharan Africa [3]. In 2017, UNAIDS estimated that in Mozambique, 18,000 (95% confidence interval [CI]: 10,000–27,000) children aged 0–14 years were newly infected with HIV [3].

In resource-limited countries, retention in PMTCT services is sub-optimal. Nearly 40% of HIV-exposed uninfected children are lost to follow-up before the age of 18 months [4, 5], and less than 43% of HEC undergo recommended early testing (before 2 months of age) [6]. These children are at higher risk of infection and death [4, 7, 8], and, without treatment, half of HIV-positive children may die before 2 years of age [8]. Identification of HEC as well as early diagnosis and initiation of ART are crucial to reducing HIV-related mortality rates in children [8].

In 2018, Mozambique accounted for 10% of global MTCT worldwide [9], and national estimates from SPECTRUM and from a population-based survey showed MTCT of 15 and 12.6%, respectively [10, 11]. SPECTRUM is a mathematical model recommended by WHO to measure PMTCT program success [12]; however, estimates depend on the quality of clinical and population data and assumptions used by the algorithm. Contrasting these models with data from community-based measurements within mother-child pairs is key to understanding the final MTCT rates (i.e. after cessation of breastfeeding) and the impact of PMTCT programs in context [13].

We measured the EMTCT impact indicators among HIV-exposed children in the Manhiça district in Southern Mozambique born after country implementation of Option B+. Our results complement clinic-based and model-based predictions to provide more accurate progress indicators.

## Methods

### Study area and population

This cross-sectional household survey was conducted in the Manhiça District in southern Mozambique. In this semi-rural district of the Maputo province, the Centro de Investigação em Saúde de Manhiça (CISM)‘s continuous HDSS documents vital events, including pregnancies, births, deaths, and migrations, since 1996 [14]. At the time of the study, the HDSS covered a total population of 186,000 individuals, 14% of whom were children aged < 5-years. In the District, community HIV-prevalence among women was estimated at 43.1% (95% CI: 37.6–48.5%) and 29.4% (95% CI: 26.7–32.0%) [15] for pregnant women attending antenatal consultations. This study included all six district administrative posts: Manhiça, 3 Fevereiro, Ilha Josina, Calanga, Maluana, and Xinavane.

The Manhiça District has 15 health centers, one rural hospital, and one referral district hospital where HIV care is offered free of charge and where Option B+ is recommended. After birth, HEC initiate 6 weeks of nevirapine, regardless of whether they were exclusively breastfed, and were tested by polymerase chain reaction (PCR; between 4 weeks and 9 months of age) and by rapid diagnostic test and confirmatory PCR (after 9 months of age). A final HIV test was performed at 18 months or 2 months after the end of breastfeeding following age-appropriate testing. Exclusive breastfeeding was recommended during the first 6 months and complementary breastfeeding during the first year [16].

### National age-appropriate HIV-testing algorithm

The national algorithm for adults and children (aged > 18 months) uses two rapid antibody tests (Determine and Unigold) in series. Documented known HIV-positive individuals are not re-tested, but those who do not know their status or self-report being HIV-negative are tested according to the algorithm. Dried blood spot samples of children aged < 18 months are laboratory tested via HIV cDNA PCR, which detects proviral DNA. Non-reactive specimens are considered HIV-negative. Reactive specimens receive a second confirmatory HIV DNA PCR. A child with positive PCR and positive confirmatory PCR results is considered HIV-positive. In the case of discordant results, a second dried blood spot sample is collected, and a third PCR test is conducted. HIV testing is accompanied by pre-test and post-test counseling, and anyone who tests positive is referred for HIV care and treatment [17].

### Study procedures and definitions

We used simple random selection to identify 5000 children born alive within the 4 years before study implementation (September 1, 2013–October 31, 2017) from a list of mothers aged > 14 years and residing in the HDSS area. A household visit was conducted between October 2017 and April 2018, and consenting mothers/caregivers completed a study-specific questionnaire. For each participant, three household visit attempts were made before defining the status as absent. If the mother was not available (due to absence, migration, or death), the child’s main caregiver provided informed consent and completed the survey. Following the National population-based survey methodology [10], for all participants (mothers and children aged > 18 months) found HIV-positive during the study visit (through documentation or new diagnosis during the household visit), a laboratory confirmation was conducted through Geenius HIV-1/2 confirmatory assay. Mother and child HIV-status was determined through documentation, age-appropriate testing, laboratory confirmation, or verbal autopsy. For those children whose mother was not available and the main caregiver provided consent, age-appropriate testing was also offered. All methods were carried out in accordance with relevant guidelines and regulations.

Probable HIV-positive included mothers or children who reported a previous HIV-positive result and without a confirmatory test result after the household visit, those whose confirmatory tests had indeterminate results, or mothers or children who had died with retrospective verbal autopsy suggesting a HIV-positive status. Confirmed HIV-positive included mothers or children with documentation of a previous HIV-positive result or those with a HIV-positive result and a positive confirmatory test after the household visit. Children out of the MTCT services included known HEC who never tested for HIV before the study visit.

### Data collection and management

The study-specific questionnaires included information on the pregnancy, history of previous HIV-testing for both mother and child, and sociodemographic information. Data from the home visits were directly collected in tablets using Open Data Kit software 1.4 [18] and were uploaded into a database in Research Electronic Data Capture (REDCap) at the end of the day [19]. Verbal autopsy interviews, adapted from the 2012 and 2016 WHO verbal autopsy sample questionnaire, were routinely conducted by CISM-trained laypersons as part of the HDSS, and results were analyzed using InterVA-M software, following the InterVA-4 model. Moreover, in all district health centers, since the day of HIV diagnosis, routine patient-level HIV clinical data are prospectively entered into an electronic patient tracking system, a Microsoft access database co-managed by the Ministry of Health and other stakeholders, where each participant had a unique numeric identifier that allows follow-up through the continuum of care. After data collection and extraction, the three databases were merged to construct the final study dataset for analysis.

### Statistical analysis

We conducted descriptive analysis stratified by child HIV-serostatus, assessing proportions by Pearson and Fisher exact chi-square tests. HIV-prevalence and transmission were calculated through two different methods: naïve, which excluded mothers and children with unknown HIV-status from the denominator, and adjusted for missing HIV-status by multiple imputation chained equation (MICE). Following MICE procedures and assuming data were missing at random, 30 cycles of imputation were generated from the missing survey data, including age, education, marital status, religion, and neighborhood. The 30 prevalence estimates were combined according to Rubin rules [20]. Fold-change in prevalence between naïve and adjusted values were calculated by dividing the adjusted by the naïve estimate. Case rate of new pediatric HIV infections was calculated as the number of children exposed to HIV per 100,000 live births multiplied by the MTCT. Logistic regression was performed to assess factors associated with children with unknown HIV status and with a late infant HIV diagnosis. Variables with *p* < 0.2 on univariate analysis were included in multivariable analysis. Fine and Gray model was applied to estimate retrospectively the cumulative mortality rate and identify risk factors among participants over the 54 months preceding the study visit. Cumulative incidence estimation in the presence of competing risks was calculated following Coviello et al. [21]. Hazard ratios of the sub-distribution (sHR) and the corresponding 95% CIs were used as a measure of association. Statistical analyses were performed using Stata 15 [22].

## Results

### Study profile and baseline characteristics

**Fig. 1:**
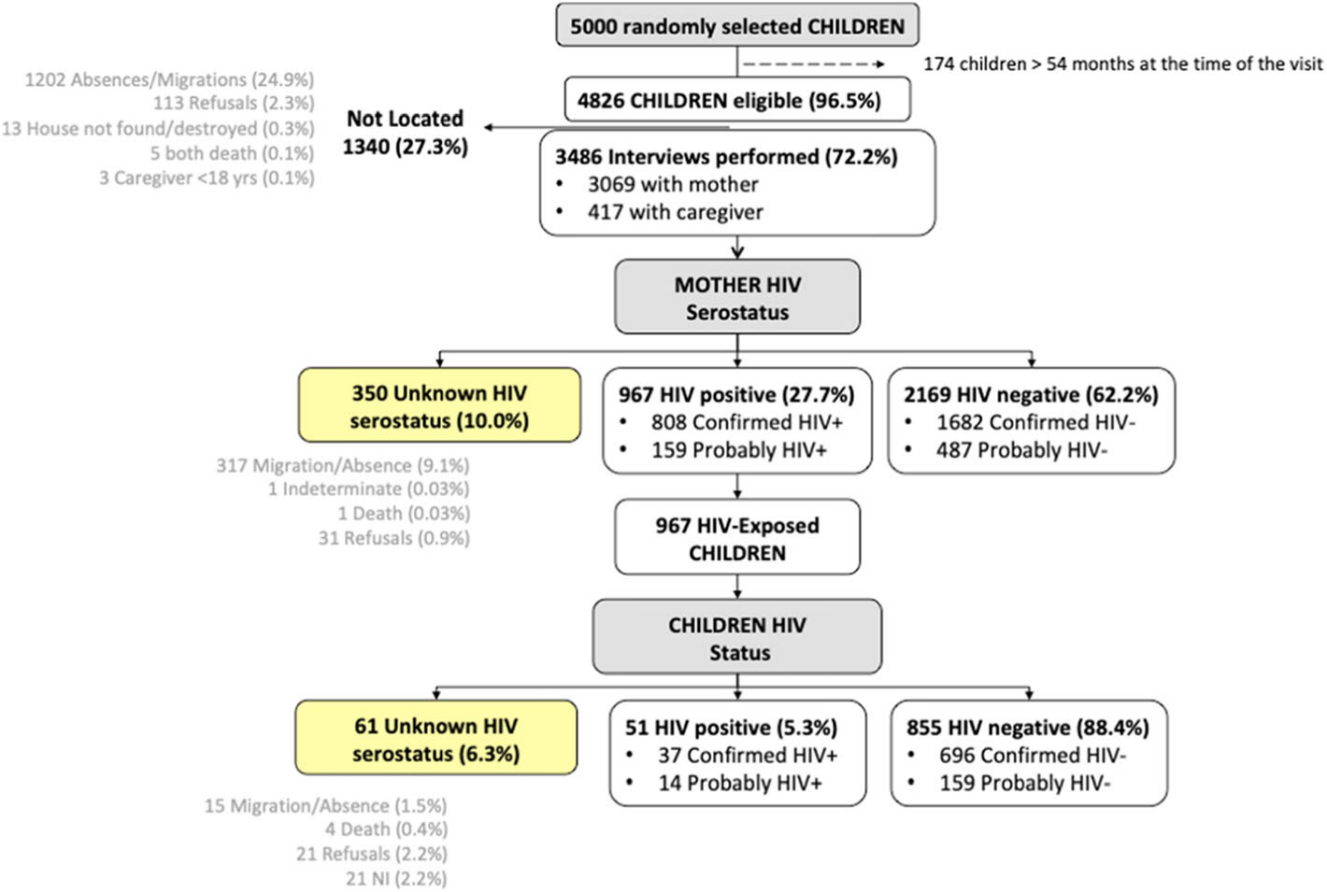
Study profile of 5000 mother-child pairs randomly selected for the study of mother-to-child transmission of HIV. Percentages are calculated over the previous parent box. Definitions: Probably HIV+ included participants (i) who reported a previous HIV-positive result and without a confirmatory test result (ii) those with indeterminate confirmatory tests result or (iii) those who had died whose verbal autopsy suggested an HIV-positive status. Abbreviation: NI, no information

Among the mother-child pairs randomly selected from the HDSS (Fig. 1), 4826 children were aged < 54 months at time of visit. Of those, 3486 (72.2%) mother/caregiver-child pairs (MCCP) participated. In most of the interviews, the respondent was the biological mother of the selected child, while 12.0% of mothers were not available (Fig. 1), and the child’s caregiver completed the interview.

We identified 967 mothers and 51 children as probable or confirmed HIV-positive. An additional 350 (10.0%) mothers and 61 (6.3%) HEC had an unknown serostatus. Migration and absence during the study period were the main reasons for the mothers’ unknown serostatus.

Baseline characteristics of HEC did not differ by child’s serostatus for most variables (Table 1); however, a significantly (*p* < 0.001) lower proportion of HIV-positive children (56.9%) had received nevirapine prophylaxis compared to HIV-negative children (82.8%) or children with unknown status (80.3%). From birth until the survey, HIV-positive children (76.5%) had more outpatient hospital visits than did HIV-negative children (55.2%) or those with unknown serostatus (52.5%; *p* = 0.01) as well as more hospital admissions (25.2, 6.0, and 6.6%, respectively; *p* < 0.001). The median duration of breastfeeding in HEC was 12 months (interquartile range [IQR], 7.1–16.0) and significantly lower than median duration among HIV-unexposed children (for further information on breastfeeding among this cohort see Fernández-Luis et al. [23]).

**Table 1:** Baseline characteristics of HIV-exposed children (*n* = 967) by children’s serostatus in Manhiça District, Mozambique

		UNKNOWN STATUS (***N*** = 61)		HIV POSITIVE (***N*** = 51)		HIV NEGATIVE (***N*** = 855)	
Characteristics		N		N	%	N	
**Median age at delivery in years (mother) (IQR)**		30.7 (23.2–34.0)		25.9 (21.7–33.0)		28.7 (23.6–33.4)	
**Mother age at delivery (years)**	**< 19**	5	8.2%	8	15.7%	92	10.8%
	**20–24**	15	24.6%	11	21.6%	171	20.0%
	**25–34**	31	50.8%	27	52.9%	463	54.2%
	**> 35**	10	16.4%	5	9.8%	129	15.1%
**Mother alive**	**Yes**	59	96.7%	50	98.0%	847	99.1%
	**No**	2	3.3%	1	2.0%	8	0.9%
**Educational level**	**No education**	7	11.5%	8	15.7%	174	20.4%
	**Basic**	42	68.9%	36	70.6%	596	69.7%
	**Medium/High**	12	19.7%	7	13.7%	72	8.4%
	**NI**	0	0.0%	0	0.0%	13	1.5%
**Marital status**	**Single**	6	9.8%	3	5.9%	112	13.1%
	**Married**	48	78.7%	41	80.4%	604	70.6%
	**Divorced/Widowed**	7	11.5%	7	13.7%	139	16.3%
**Income**	**Domestic**	3	4.9%	1	2.0%	6	0.7%
	**No fixed salary/agriculture**	35	57.4%	24	47.1%	438	51.2%
	**Fixed Salary**	23	37.7%	26	51.0%	409	47.8%
	**NI**	0	0.0%	0	0.0%	2	0.2%
**Type of administrative post (habitants)**	**Big cities (> 15,000)**	20	32.8%	21	41.2%	353	41.3%
	**Medium posts (5000)**	34	55.7%	26	51.0%	401	46.9%
	**Small posts (< 5000)**	7	11.5%	4	7.8%	101	11.8%
**ANC visits**^a^	**No**	1	1.6%	3	5.9%	14	1.6%
	**Yes**	60	98.4%	48	94.1%	841	98.4%
**Mother newly HIV diagnosed**	**No**	57	93.4%	48	94.1%	801	93.7%
	**Yes**	4	6.6%	3	5.9%	54	6.3%
**Parity at the time of the child’s birth**	**1**	10	16.4%	9	17.6%	117	13.7%
	**2**	13	21.3%	14	27.5%	157	18.4%
	**3 or more**	38	62.3%	28	54.9%	581	68.0%
**Other children who died after born alive (including stillbirths)**	**No**	48	78.7%	33	64.7%	596	69.7%
	**Yes**	13	21.3%	18	35.3%	255	29.8%
	**NI**	0	0.0%	0	0.0%	4	0.5%
**Preterm birth**	**Yes**	4	6.6%	3	5.9%	72	8.4%
	**No**	31	50.8%	24	47.1%	459	53.7%
	**NI**	26	42.6%	24	47.1%	324	37.9%
**Place of birth**	**Peripheric Health Unit**	44	72.1%	34	66.7%	527	61.6%
	**Manhiça District Hospital**	12	19.7%	14	27.5%	270	31.6%
	**Home/On the way**	4	6.6%	1	2.0%	37	4.3%
	**NI**	1	1.6%	2	3.9%	21	2.5%
**Age at visit (months)**	**< 6**	2	3.3%	0	0.0%	25	2.9%
	**6–12**	8	13.1%	10	19.6%	111	13.0%
	**12–18**	10	16.4%	6	11.8%	131	15.3%
	**18–24**	13	21.3%	8	15.7%	147	17.2%
	**> 24**	28	45.9%	27	52.9%	441	51.6%
**Median age at visit in months (IQR)**		22.8 (16.1–34.4)		25.3 (13.8–37.3)		24.6 (15.7–36.5)	
**Sex**	**Male**	23	37.7%	22	43.1%	434	50.8%
	**Female**	38	62.3%	29	56.9%	421	49.2%
**Child breastfeed**	**Yes**	57	93.4%	51	100.0%	831	97.2%
	**No**	4	6.6%	0	0.0%	24	2.8%
**Median time of brestfeeding (IQR)**		10.2 (6.0–15.0)		12.1 (9.1–18)		12.0 (7.0–16.0)	
**Order of birth**	**<= 3**	31	50.8%	30	58.8%	477	55.8%
	**> 3**	30	49.2%	21	41.2%	378	44.2%
**Child born in Mozambique**	**No**	1	1.6%	2	3.9%	21	2.5%
	**Yes**	60	98.4%	49	96.1%	834	97.5%
HIV prophylaxis (syrup)*	**Yes**	49	80.3%	29	56.9%	708	82.8%
	**No**	2	3.3%	7	13.7%	23	2.7%
	**NI**	10	16.4%	15	29.4%	124	14.5%
**Updated age-appropriated vaccines**	**Yes**	16	26.2%	18	35.3%	308	36.0%
	**No**	21	34.4%	16	31.4%	294	34.4%
	**NI**	24	39.3%	17	33.3%	253	29.6%
Child with OPD visits*	**No**	29	47.5%	12	23.5%	383	44.8%
	**Yes**	32	52.5%	39	76.5%	472	55.2%
Median number of OPD visits (IQR)*		1 (0–3)		2 (1–5)		1 (0–4)	
Number of OPD visits*	**None**	29	47.5%	12	23.5%	383	44.8%
	**1–2**	15	24.6%	16	31.4%	184	21.5%
	**3–5**	12	19.7%	11	21.6%	142	16.6%
	**≥ 6**	5	8.2%	12	23.5%	146	17.1%
Child with INPD visits*	**No**	57	93.4%	38	74.5%	804	94.0%
	**Yes**	4	6.6%	13	25.5%	51	6.0%
Median number of INPD visits (IQR)*		0.1 (0–0)		0.3 (0–1)		0.1 (0–0)	
Number of INPD visits*	**None**	57	93.4%	38	74.5%	804	94.0%
	**1**	4	6.6%	10	19.6%	42	4.9%
	**2**	0	0.0%	3	5.9%	9	1.1%

*Abbreviations*: *IQR* interquartile range, *ANC* antenatal consultations, *FU* follow-up, *OPD* out-patient department, *INPD* in-patient department, *NI* no information available. ^a^ANC Visits: participants were asked if they performed at least one antenatal consultation during their pregnancy

* *p* < 0.05, ttest or Fisher’s exact test, as appropiate

### Maternal HIV-prevalence and EMTCT impact indicators

The estimated unadjusted community HIV-prevalence among women with a live birth in the previous 4 years was 30.8% (95% CI: 29.2–32.5%; Table 2). However, after adjustment for mothers with unknown HIV serostatus, the estimate of HIV-prevalence increased significantly to 37.6% (95% CI: 35.8–39.4%). Specifically, adjusted HIV-prevalence doubled in mothers aged < 19 years (from 8.0 to 19.1%) and increased 1.5 times- in mothers aged < 25 years (from 21.3 to 31.8%; Table 2) as compared to unadjusted prevalence.

**Table 2:** Community HIV-prevalence in mothers and vertical transmission among HIV-exposed children for interviewed mother/caregiver-child pairs (*n* = 3486)

AGE GROUP	HIV PREVALENCE & TRANSMISSION				
	Naive^a^	95% CI	MICE^b^	95% CI	Fold-change in prevalence
MOTHER (years)	30.8%	29.2–32.5	37.6%	35.8–39.4	1.2
< 19	8.0%	5.9–10.9	19.1%	15.7–22.6	2.4
20–24	21.3%	18.7–24.1	31.8%	28.7–34.9	1.5
25–34	40.4%	37.7–43.1	44.8%	42.0–47.5	1.1
> 35	43.3%	39.0–47.6	47.4%	43.2–51.6	1.1
Child TOTAL	5.6%	4.3–7.3	4.4%	3.1–5.7	0.8
< 24 months	5.5%	3.7–8.1	4.9%	2.9–6.9	0.9
≥ 24 months	5.8%	4.0–8.3	4.0%	2.4–5.5	0.7

^a^For naive calculations, in both cases (mother and children), individuals with unknown serostatus were excluded. Thus, the denominators for naive community HIV prevalence were *n* = 3136 for mothers and *n* = 906 for children. ^b^Mothers and children with unknown serostatus were treated as missing values, and estimates of prevalence and transmission were adjusted by multiple imputation (MICE). In children, the selected age to stratified the cohort was 24 months to compare our results with national data [10]. Fold-change in prevalence between naïive and adjusted values was calculated following the definition in the Methods section. *Abbreviations*: *CI* confidence interval, *MICE* multiple imputation chained equation

HIV transmission in HEC, adjusted for unknown maternal and child serostatus was 4.4% (95% CI: 3.1–5.7%) among children < 48 months and 4.9% (95% CI: 2.9–6.9%) among those aged < 24 months (Table 2). The cumulative rate of new pediatric HIV infections occurring between birth was estimated at 1654 new infections per 100,000 live births.

### Early infant testing and associated factors

Of the 967 mothers living with HIV (MLHIV) (see Figure S1), 708 (73.2%) received the diagnosis before the delivery of the child and 169 (17.5%) after delivery; for 90 (9.3%) of the MLHIV, the date of diagnosis was unknown. Although 95.6% (677/708) of the HEC had been tested for HIV at some point before our survey, only 69.1% (489/708 [95% CI: 65.5–72.5%]) had been tested in the first 2 months of life.

Risk factors for not having an HIV test were assessed among HEC of mothers infected before delivery (Table 3). Calculations excluded children who died in the first 2 months of life and for whom there was not enough time for initial testing. Multivariate logistic regression analysis showed that those children who did not receive HIV prophylaxis during their first 6 weeks of life were 36.45 (95% CI: 5.09–260.82) times more likely to be out of the MTCT services than those who did. Variables highly associated with a child never testing such as educational level and not attending antenatal consultations [ANC]) were not included in the multivariable model due to collinearity. All HEC whose mothers had medium/high education had been previously tested. All HEC whose mothers self-reported not attending ANC had not previously been tested (See Figure S2 for further information regarding the PMTCT retention in care among found mothers).

**Table 3:** Analysis of factors associated with never being HIV-tested before the study recruitment for HEC

		OR	95% CI	***p*** value	aOR	95% CI	***p*** value
**Median age at delivery in years (mother)**		**0.94**	**0.87–1.02**	0.141*	0.92	0.83–1.01	0.094
**Mother age at delivery (years)**	**< 19**	ref	–	–			
	**20–24**	1.03	0.19–5.47	0.975			
	**25–34**	0.61	0.13–2.92	0.539			
	**> 35**	0.25	0.02–2.82	0.262			
**Educational level**	**None**	ref	–	–			
	**Some**	0.55	0.19–1.60	0.275			
**Marital status**	**Single**	**ref**	**–**	**–**	ref	**–**	**–**
	**Married**	**0.43**	**0.13–1.39**	0.158*	0.43	0.11–1.75	0.241
	**Divorced/Widowed**	**0.62**	**0.13–2.87**	**0.544**	0.23	0.03–1.87	0.171
**Income**	**No fixed salary**	ref	–	–			
	**Fixed Salary**	0.79	0.30–2.10	0.634			
**Type of administrative post (habitants)**	**Big cities (> 15,000)**	ref	–	–			
	**Medium posts (5000)**	1.22	0.48–3.89	0.721			
	**Small posts (< 5000)**	2.03	0.74–9.89	0.324			
**Parity**	**1**	ref	–	–			
	**2**	2.85	0.33–24.9	0.344			
	**3 or more**	1.72	0.22–13.56	0.606			
**Preterm birth (< 37 weeks)**	**No**	**ref**	**–**	**–**	ref	**–**	**–**
	**Yes**	**5.72**	**1.58–20.73**	0.008*	3.83	0.79–18.04	0.093
**Place of birth**	**Peripheric Health Unit**	**ref**	**–**	**–**	ref	–	–
	**Manhiça District Hospital**	**0.32**	**0.70–1.45**	0.138*	0.23	0.04–1.23	0.086
	**Home/On the way**	**3.13**	**0.65–15.19**	0.156*	2.64	0.40–17.29	0.312
**Sex**	**Male**	ref	–	–			
	**Female**	1.16	0.44–3.05	0.760			
**Child breastfeed**	**Yes**	**ref**	**–**	**–**	ref	–	–
	**No**	**4.62**	**0.98–21.67**	0.052*	4.73	0.65–34.57	0.126
**Order of baby**	**<=3**	ref	–	–			
	**> 3**	0.61	0.22–1.67	0.333			
**Child born in Mozambique**	**No**	**ref**	**–**	**–**	ref	–	–
	**Yes**	**0.10**	**0.02–0.51**	0.006*	0.16	0.02–1.48	0.107
**HIV prophylaxis (syrup)**	**Yes**	**ref**	**–**	**–**	**ref**	**–**	**–**
	**No**	**29.90**	**6.84–130.71**	< 0.001*	**36.45**	**5.09–260.82**	< 0.001*

This analysis was performed among HIV-exposed children, excluding those who died before 2 months of age, in Manhiça District, Mozambique (*n* = 694). Owing to adjustments for missing data with multiple imputation, no absolute numbers were included in the table, only proportions. *Abbreviations*: *CI* confidence interval, *OR* odds ratio, *aOR* adjusted odds ratio. * Variable with p < 0.2 on univariate analysis included in multivariable analysis

### Child mortality

**Fig. 2:**
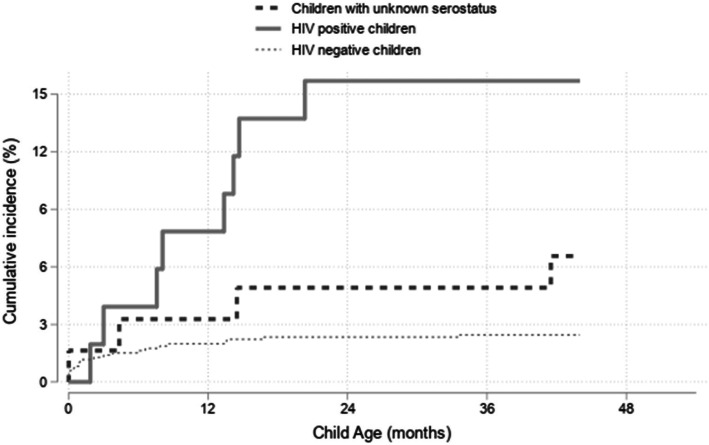
Cumulative incidence of mortality, in the presence of competing risk, among HEC and stratified by children serostatus. Competing risk included migration outside of the health and demographic surveillance system

The cumulative incidence of death in the overall cohort was 41.6/1000 live births in the 54 months before the survey. Figure 2 shows the cumulative incidence of mortality among HEC, by serostatus and with the competing risk of migration. Among HEC and after adjusting by sex and birth order (Fig. 2), risk factors associated with death included HIV-positivity (asHR, 7.2 [95% CI: 3.3–15.9]; *p* < 0.001) and birth order > 3 (asHR, 3.1 [95% CI: 1.5–6.5]; *p* = 0.003). Moreover, close to the margin of significance (*p* = 0.055), children with unknown serostatus were 2.8 times (95% CI: 1.0–8.0) more likely to die than HIV-negative children.

Of the 149 children who died, 33 had been exposed to HIV. Cause of death was determined by verbal autopsy for 55.0% of the deceased children (see Table S1). Neonatal complications (30.5%; e.g., neonatal pneumonia, congenital malformations, sepsis, prematurity, and birth asphyxia), HIV-related causes (24.4%), and acute respiratory infections including pneumonia (17.1%) were the leading causes of death, followed by malaria (8.5%), non-communicable diseases (7.3%; mainly malnutrition), and diarrhea (6.1%). Median age of children who died of HIV-related causes was 14.0 months (IQR, 7.8–16.3); for those with unknown cause of death, median age was 7.7 months (IQR, 1.7–15.9); overall, median age at death was 6.8 months (IQR, 0.9–14.2).

## Discussion

Our findings show that in the district of Manhiça, approximately one third of women who had had a child in the previous 4 years were living with HIV. Although the mother-to-child transmission was below 5%, the case rate of new pediatric HIV infections was 1654 per 100,000 live births, which exceeds the WHO target rate of 50 new HIV infections per 100,000 required to eliminate MTCT. The proportion of HEC our cohort that have been tested for HIV at some point before the study visit (thus before the age of 54 months) was close to 96%, however, early infant testing only occurred in 69.1% of these children. The main factors associated with not testing were lack of ANC for the mother and lack of infant HIV-prophylaxis in the first weeks of life. The cumulative overall child mortality rate in our cohort was 41.6 per 1000 live births; with HIV-infection and later birth order significantly associated with death. Neonatal complications, HIV, and pneumonia were the main causes of death in our pediatric cohort.

Our results show that the high HIV-prevalence in women who had a baby in the last 4 years, especially in women aged > 25 years, impedes reaching the EMTCT impact target of < 50 new HIV infections per 100,000 live births even with an MTCT rate < 5%. Although great improvements in MTCT rates have been achieved, the case rate of new infections continues to exceed the WHO target and is similar to previous estimates [24]. As Goga et al. stated, in settings where HIV-prevalence in women is ≥10%, the case rate objective < 50 is not achievable [25]. With increasing access to lifelong ART, the HIV-prevalence in women of childbearing age may remain high for the next decade, even if we succeed in dramatically decreasing the rate of new infections in these women. Our findings thus support the global need of new monitoring targets adapted to high HIV-prevalence settings. Indeed, after adjusting for missing data, our estimates of prevalence in young women doubled, strongly suggesting that clinical and population-based estimates of prevalence may be underestimated in this population. Young women may be disproportionately left behind by the health system when monitoring the progress toward the global elimination of the HIV epidemic. HIV-testing for women of reproductive age is mainly performed during pregnancy via ANC services; however, several studies, especially in sub-Saharan Africa, have demonstrated that many young women do not access ANC services [26, 27]. Additionally, previous results at the national level show that close to 65% of pregnant women are lost to follow-up before completing the four recommended ANC visits [10], commonly due to migration, as described by our group and studies in other neighboring regions, such as KwaZulu Natal, where close to 50% of young women reported high rates of mobility [13, 28]. Migration and lack of access to ANC makes this vulnerable group less likely to be aware of their HIV-positive status and thus at higher risk of transmitting HIV to their children.

Our community-based MTCT estimates of 4.4% contrast with the national vertical transmission estimated at 15% by SPECTRUM and 12.6% in the national population-based survey conducted in 2015 [10, 11]. Both methods estimated MTCT at the national, whereas we have estimated at the district level. In addition, the accuracy of the SPECTRUM estimates depends on the availability and quality of the data used, however, in most resource-limited countries, clinical follow-up at health facilities is recorded on paper, and electronic monitoring tracking systems only include patients after their HIV-positive diagnosis, and no information regarding patients’ relatives is registered. One of the main limitations of this model is that in the absence of quality data, the estimates generated through the model nay not represent the true situation of the epidemic in the country. After maternal HIV-diagnosis, follow-up of HEC is the second most crucial challenge to monitoring MTCT. Our findings highlight the importance of collecting community-based data and conducting analysis at subnational-level that allows to track mother-child pairs, link their clinical data and obtain more accurate and updated estimates at regional level that could be used to adjust national ones. The INDEPTH network of HDSS has shown the contribution of community-based demographic surveillance to improving estimates of malaria, fertility rates and death rates among others. Mozambique has three demographic HDSS which could be leveraged to help adjust program implementation indicators that might otherwise be overestimated [12, 13, 29].

Lack of ANC uptake in mothers and subsequent lack of PMTCT services among HEC were found to be the main factors associated with late HIV-testing (2 months after birth). In our cohort, coverage of HIV testing among HEC was high (96%), compared to national estimates where only half of HEC were tested after 2 years of age [10]; however timely diagnosis occurred in less than 70% of them. Early infant diagnosis and early treatment has been widely associated with decreased child mortality [10]. The lack of sustained ART for HIV-positive children, due to low testing and/or ART coverage, was shown across nine sites in South Africa to be largely responsible for rates of mortality. We found that children living with HIV are seven times more likely to die than HIV-negative children, and our findings support others that HIV continues to be the second leading cause of death among children aged < 5 years. Effective interventions that identify HEC and increase HIV testing, including mHealth, and healthcare strategies to improve the quality of care at facilities could help improve outcomes for HEC in high-HIV burden settings [30–33].

Community interventions that promote health education among adolescent mothers both during the prenatal and postnatal period have substantially decreased infant mortality rates and increased ANC uptake [34]. Strategies such as phone reminders, personal counseling, or home-visits may help re-engage women lost from ANC and maternal and child health care and ensure that those women who initially tested HIV-negative during pregnancy or breastfeeding [35] receive repeat testing [36, 37]. These strategies in parallel with linking mother and child clinical information would optimize the impact of PMTCT programs decreasing the number of new pediatric HIV infections and facilitating the timely diagnosis of HIV and treatment initiation among HEC. Furthermore, trans-border strategies could help facilitate clinical follow-up of young populations and ensure their health coverage to help control the HIV epidemic [38–40].

Strengths of our study include the HDSS platform, which not only records information on migration and deaths but also links the information of mother-child pairs and facilitates accurate HIV-prevalence estimates and MTCT rates among HEC at community level. Our study had several limitations. First, even though we used different sources to determine the HIV status of all participants, 10% of the mothers and 6.3% children still did not know their serostatus. To account for these missing values, we applied multiple imputation assuming that HIV status was missing at random. However, there is evidence that these values may not be missing random; thus, we cannot exclude selection bias [41–43]. Second, our analysis is limited by the small sample size of the HIV-positive pediatric cohort and encourages caution as to the magnitude of associations of risk factors and not receiving timely testing.

## Conclusions

In areas with high prevalence of HIV, even with an MTCT rate < 5%, the EMTCT impact target of < 50 new HIV infections per 100,000 live births may not be achievable. Indicators to galvanize PMTCT programs should be designed specifically for a spectrum of HIV-prevalence values > 10% in women of childbearing age. There is a need for novel models that also estimate the maximum incidence of new HIV infections in young women who will be bearing children in the next decade in order to truly reach the EMTCT targets. Ensuring that all pregnant women attend ANC services, especially adolescents and young mothers, could help reduce HIV incidence and increase ART coverage. Ensuring that all HIV-positive women know their status and that their children receive the necessary preventive care in a timely manner could help prevent HIV transmission and decrease mortality in CLHIV. Finally, quality community-level data are needed to adjust prevalence data in women of childbearing age as well as to adjust estimates on prevention of mother-to-child HIV transmission.

## Supplementary Information

**Additional file 1: Figure S1**. HIV testing history among seropositive women in our cohort and their children who participated in a study of mother-to-child transmission of HIV in Manhiça District, Mozambique. **Table S1**. Leading causes of death among deceased children (*n* = 82) in a study of mother-to-child transmission of HIV in Manhiça District, Mozambique. Children with unknown cause of death due to absence of verbal autopsy were excluded from the denominator. **Figure S2**. HIV testing history and PMTCT retention among women found at home who participated in a study of mother-to-child transmission of HIV in Manhiça District, Mozambique.

## Data Availability

The datasets used and/or analyzed during the current study are available from the corresponding author on reasonable request.
