# Supplementary material for: Community-based progress indicators for prevention of mother-to-child transmission and mortality rates in HIV-exposed children in rural Mozambique
Source: BMC Public Health. 2021 Mar 17;21:520. doi: 10.1186/s12889-021-10568-4 (PMC7970736; doi:10.1186/s12889-021-10568-4)
Supplement: Supplementary file 1 — Additional file 1: Figure S1. HIV testing history among seropositive women in our cohort and their children who participated in a study of mother-to-child transmission of HIV in Manhiça District, Mozambique. Table S1. Leading causes of death among deceased children (n = 82) in a study of mother-to-child transmission of HIV in Manhiça District, Mozambique. Children with unknown cause of death due to absence of verbal autopsy were excluded from the denominator. Figure S2. HIV testing history and PMTCT retention among women found at home who participated in a study of mother-to-child transmission of HIV in Manhiça District, Mozambique. [file 12889_2021_10568_MOESM1_ESM.docx]

**Community-based progress indicators for prevention of mother-to-child transmission and mortality rates in HIV-exposed children in rural Mozambique**

Laura Fuente-Soro, MRes, PhD^1,2^; Sheila Fernández-Luis, MD^1,2^; Elisa López-Varela, MD, MPH, PhD^1,2^; Orvalho Augusto, MD, MPH^1^; Tacilta Nhampossa, MD, PhD^1,3^; Ariel Nhacolo, MSc^1^; Edson Bernardo, MD, MPH^4,5^; Blanca Burgueño, MD^2^; Bernadette Ngeno, MD^6^; Aleny Couto, MD^7^; Helga Guambe, MD^7^; Kwalila Tibana, MD^7^; Marilena Urso, MD^8^; Denise Naniche, MPH, PhD^1,2^.

1. Centro de Investigação em Saúde de Manhiça, Maputo, Mozambique; 2. Barcelona Institute for Global Health, Barcelona, Spain; 3. Instituto Nacional de Saúde, Maputo, Mozambique; 4. Manhiça District Health Services, Maputo, Mozambique; 5. Vanderbilt Institute for Global Health, Tennessee, USA; 6. U.S. Centers for Disease Control and Prevention, Division of Global HIV and Tuberculosis, Atlanta, USA; 7. Ministério da Saúde de Moçambique, Maputo, Mozambique; 8. U.S. Centers for Disease Control and Prevention, Division of Global HIV and Tuberculosis, Maputo, Mozambique

Corresponding author: Laura Fuente-Soro, ISGlobal, Barcelona Institute for Global Health, Barcelona, Spain, Rossello, 132, 08036, Barcelona, Spain .Tel. +34 93 227 1806 Fax, Email: [laura.delafuente@isglobal.org](mailto:laura.delafuente@isglobal.org)

Key words: **HIV, MTCT, Mozambique, Africa, mother-to-child transmission, HIV-prevalence**

**Supplementary material**


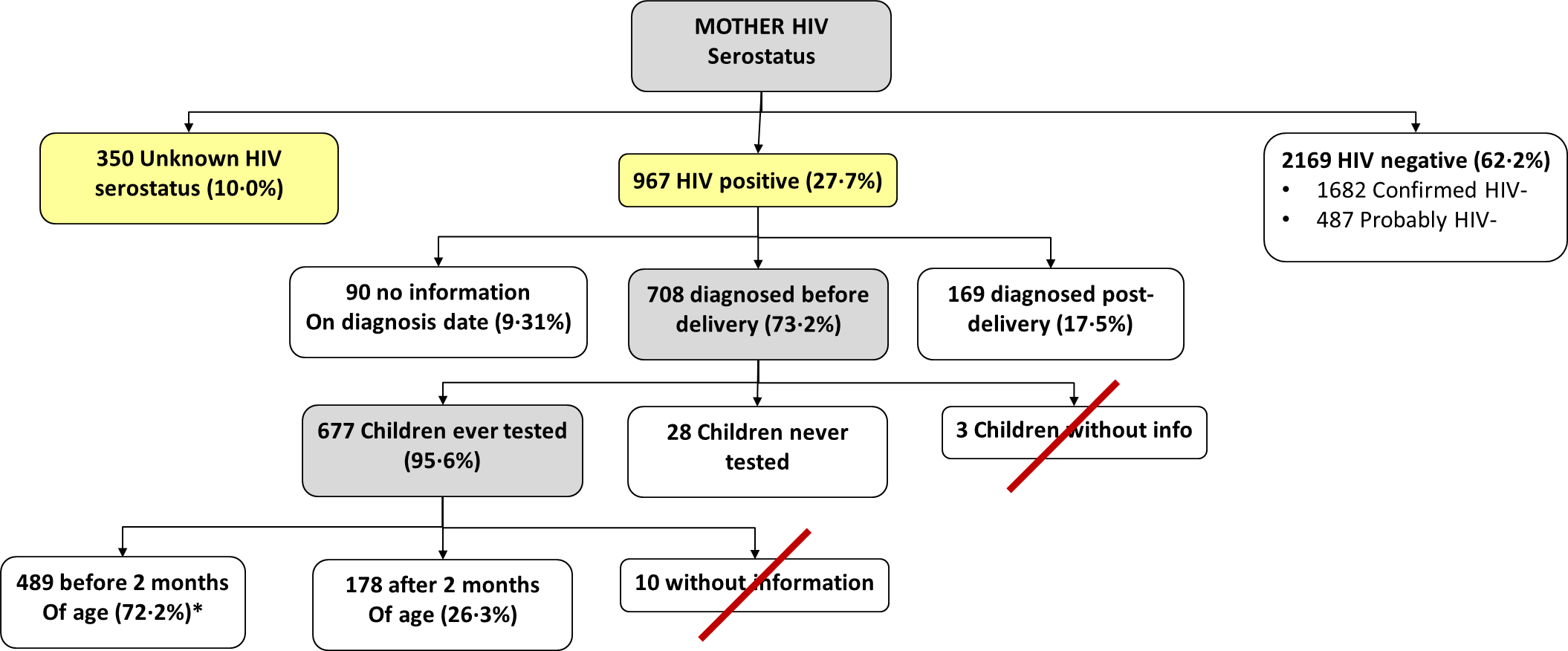


**Figure S1. HIV testing history among seropositive women in our cohort and their children who participated in a study of mother-to-child transmission of HIV in Manhiça District, Mozambique.** Percentages are calculated based on the previous step. Definitions: Probably HIV+ included participants (i) who reported a previous HIV-positive result and without a confirmatory test result (ii) those with indeterminate confirmatory tests result or (iii) those who had died whose verbal autopsy suggested an HIV-positive status. Abbreviation: NI, no information.

**Table S1. Leading causes of death among deceased children (n=82) in a study of mother-to-child transmission of HIV in Manhiça District, Mozambique. Children with unknown cause of death due to absence of verbal autopsy were excluded from the denominator**.

|  | **n** | % |
| --- | --- | --- |
| **Neonatal related causes** | 25 | 30,5% |
| **HIV related causes** | 20 | 24,4% |
| **Acute respiratory infection including Pneumonia** | 14 | 17,1% |
| **Malaria** | 7 | 8,5% |
| **Non-communicable diseases** | 6 | 7,3% |
| **DiarrheaDiarrhea** | 5 | 6,1% |
| **Other Infectious Diseases** | 4 | 4,9% |
| **External Causes** | 1 | 1,2% |
| TOTAL | 82 |  |

Causes of death were analyzed by InterV4. Neonatal causes included neonatal pneumonia, congenital malformation, prematurity, sepsis, and birth asphyxia. Non-communicable diseases included malnutrition, liver cirrhosis, and epilepsy. Other infectious diseases included pertussis, meningitis, encephalitis, and non-obstetric sepsis.


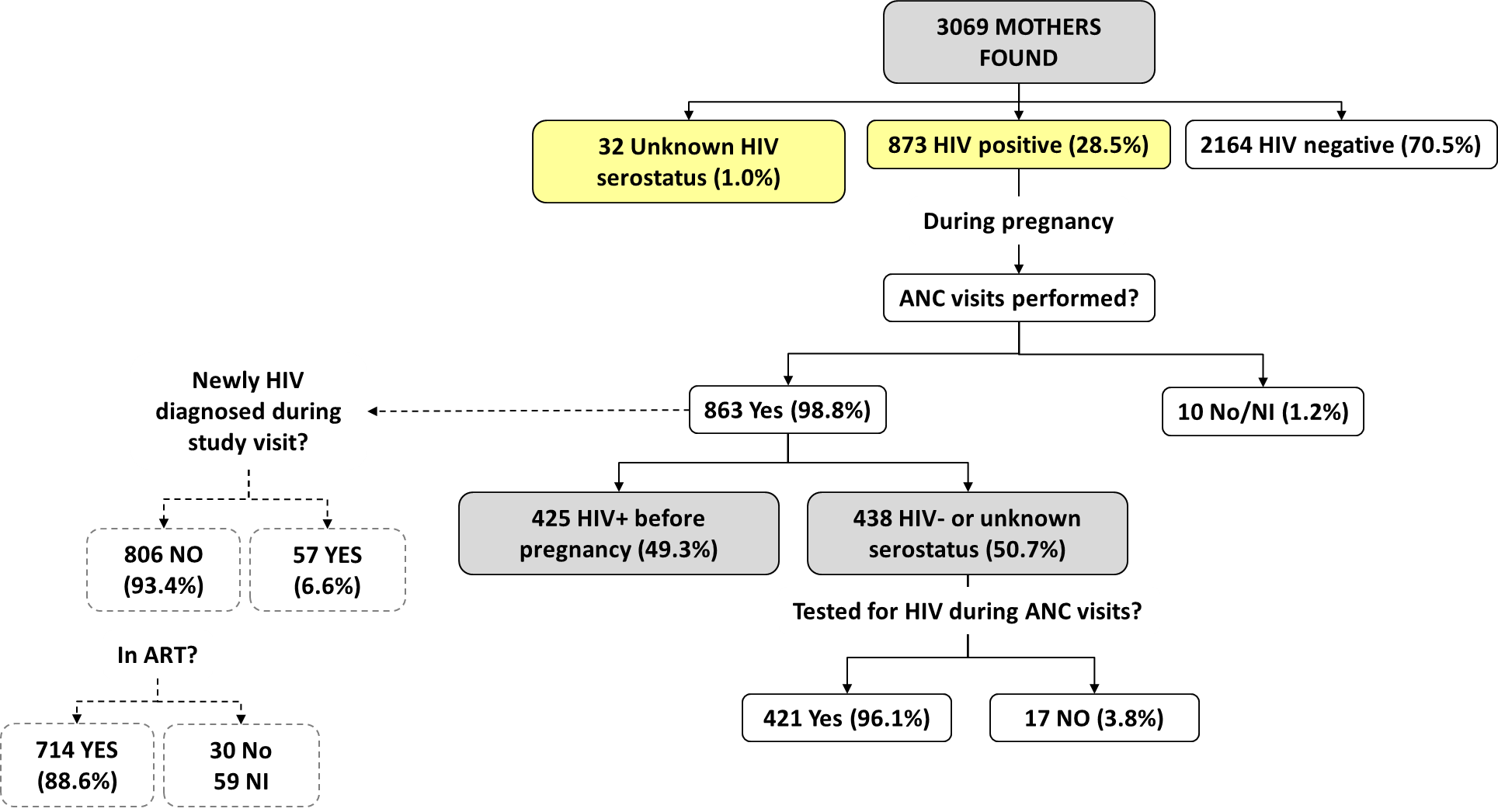


**Figure S2. HIV testing history and PMTCT retention among women found at home who participated in a study of mother-to-child transmission of HIV in Manhiça District, Mozambique.** Percentages are calculated based on the previous step. Abbreviation: NI, no information. ART, antiretroviral therapy. ANC, antenatal consultation.
